# Development of a formula for estimated glomerular filtration rate in pregnant women from physiological hyperfiltration of serum creatinine

**DOI:** 10.1038/s41598-024-57737-0

**Published:** 2024-03-27

**Authors:** Kwangjin Ahn, Taesic Lee, Jieun Kang, Seong Jin Choi, Sangwon Hwang, Dong Min Seo, Jooyoung Cho, Young Uh

**Affiliations:** 1 Department of Laboratory Medicine, Yonsei University Wonju College of Medicine, Wonju-si, Republic of Korea; 2 Department of Family Medicine, Yonsei University Wonju College of Medicine, Wonju-si, Republic of Korea; 3 Department of Obstetrics and Gynecology, Yonsei University Wonju College of Medicine, Wonju-si, Republic of Korea; 4 Department of Precision Medicine, Yonsei University Wonju College of Medicine, Wonju-si, Republic of Korea; 5 Department of Medical Information, Yonsei University Wonju College of Medicine, Wonju-si, Republic of Korea

**Keywords:** Nephrons, Computational models

## Abstract

Increased body fluids during pregnancy complicates the application of estimated glomerular filtration rate (eGFR) formulas that are based on body surface area. Furthermore, gestational renal dysfunction cannot be identified if the serum creatinine (SCr) concentration is within the non-pregnant reference interval (RI) despite inadequate pregnancy-related renal hyperfiltration. 1484 SCr measurements from 957 healthy pregnant women were collected. The average SCr value of gestational week (GW) 0–3 was the representative SCr value of non-pregnant status. While the distribution of SCr measurements varied across GWs, it was transformed into a normal distribution using the bootstrap resampling method. A polynomial linear regression method was applied to achieve a continuous and smooth transformation of values. The normally distributed SCr values of each GW were compared to the non-pregnant status, leading to the calculation of SCr hyperfiltration. The final equation, (2 − SCr (μmol/L)$/$55.25)$\times$ 103.1 $\times$ 55.25/(56.7 − 0.223 $\times$ GW − 0.113 $\times$ GW^2^ $+$ 0.00545 $\times$ GW^3^ − 0.0000653 $\times$ GW^4^), and reference intervals for both SCr and eGFR for each GW were obtained. These RIs and novel equations can be effectively used to monitor renal dysfunction in pregnant women.

## Introduction

Pregnancy causes physiological alterations including blood volume expansion due to increased cardiac output and decreased systemic vascular resistance^1,2^. Systemic vasodilation and glomerular hyperfiltration are normal hemodynamic adaptations during pregnancy^1^. The glomerular filtration rate (GFR) increases during pregnancy, whereas the serum creatinine (SCr) concentration decreases according to the degree of maternal GFR change. Consequently, the formula for estimated GFR (eGFR) based on SCr concentration cannot be accurately applied to pregnant women^3^. eGFR values may be inaccurate as urine output and body weight vary depending on the gestational week (GW), and the proportion of muscular component of the total body surface area (BSA) in pregnant women differs from that in non-pregnant women. Wang et al.^4^ analyzed that an increase in intravascular volume and body water during pregnancy could overestimate the calculated BSA. Its feasibility in pregnant women has not been well studied, and existing guidelines exclude the application of eGFR formulas in pregnant women^5,6^.

Accurate determination of SCr concentrations in pregnant women, according to gestational age, is crucial because application of reference intervals (RIs) designed for non-pregnant women can misinterpret adverse pregnancy outcomes or abnormal kidney function as normal. These RIs do not reflect normal physiological changes during pregnancy and hinder early diagnosis of chronic kidney disease (CKD) and appropriate treatment for complications affecting the fetus and mother. The Clinical and Laboratory Standards Institute (CLSI) recommends establishing an RI by collecting samples from a sufficient number of qualified reference individuals to yield a minimum of 120 observations for analysis^7^.

Rapid advancements in computer technology and the availability of cheaper, smaller devices have led to the development of new statistical methods. The bootstrap method, a computer-intensive resampling technique, is a statistical method^8,9^ that involves the generation of subsets of a fixed size using random sampling (with replacements) from the original data^9^. Aggregating the results allows for easy calculation of the mean or median, variance or standard deviation, and confidence intervals without assuming the original data distribution^9^. This method is valuable for estimating the RIs for small sample sizes^9,10^.

**Figure 1:**
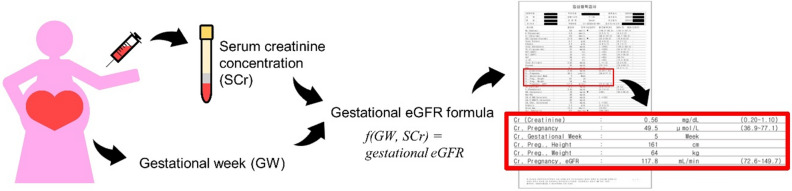
Reporting scheme for gestational SCr RIs and estimated glomerular filtration rate (eGFR) with gestational eGFR value. When an obstetrician ordered SCr measurement and recorded the GW of a pregnant woman, the gestational eGFR was automatically calculated, and the status of SCr concentration and eGFR were verified. It's obvious that the tools developed in this study have been applied in a laboratory information system, and the results have been reported completely.

This study involved two main tasks. First, a database table was created to manage GW-specific SCr concentrations and eGFR normal RIs using a laboratory information system (LIS). Second, a real-time reporting system for eGFR was established based on the data from electronic medical records (EMRs) (Fig. 1).

## Methods

### Data collection

Relevant data between January 2010 and December 2020 were collected from EMRs and LIS at Wonju Severance Christian Hospital (WSCH) and automatically decoded. The analysis included women who had singleton or multiple births at > 38 weeks of gestation, were aged 16–50 years at the time of delivery, and had at least one SCr measurement during pregnancy. We previously analyzed the SCr concentrations in 4,004 pregnant women to predict adverse pregnancy outcomes^11^. EMR extracted data were reviewed by an expert from the Department of Medical Information, and LIS data were reviewed by laboratory medicine specialists. Finally, obstetricians confirmed the absence of adverse pregnancy outcomes in the selected reference group through a manual record review, especially, underlying disease and pregnancy adverse outcomes that contained acute renal disease, chronic renal disease, and hypertensive disorders which affected renal function were excluded. The final 957 pregnant women were chosen as reference individuals (Supplementary Fig. S1). For cases in which multiple SCr measurements were performed during pregnancy, only the initial SCr results were included. If a patient had multiple measurements of SCr in different GWs, all the initial SCr values representing each GW were included. SCr concentrations were measured using the Cobas® c 702 (Roche Diagnostics, Switzerland), Modular DPE analyzer (Roche Diagnostics), Vitros FS 5.1 (Ortho Clinical Diagnostics, Raritan, NJ, USA), Vista 1500 (Siemens Healthineers, Erlangen, Germany), and Atellica CH 930 analyzer (Siemens Healthineers). SCr concentrations were analyzed by conversion from mg/dL to μmol/L.

### Assessment of suitability for creating GW-specific SCr RI

CLSI recommends that utilizing two approaches to establish a 95% confidence interval (CI) for each subclass (e.g., age group and sex): either selecting qualified reference individuals or including a sufficient number of participants, with a recommended minimum of 120 subjects^7^. To address such challenging situations, the latest CLSI guidelines suggest that if it is difficult to collect 120 reference individuals for each subclass to establish the RI, it is permissible to construct the RI with a minimum of 20 individuals^7,12^. CLSI recommends using a non-parametric method to establish RIs regardless of the distribution of values representing normal individuals. That is to select the RI as the range between the 2.5th and 97.5th percentiles to include the central 95% CI^7^. The RI constructed in this way should be confirmed for the practical usability, defined as same or more than 95 percent of results falling within the RI when at least 20 normal individuals’ results were input.

### Bootstrap resampling

If a number of samples were much less for developing RIs, bootstrap resampling method was adapted for resolving this limitation. The initial step of resampling was divided whole data into several class having suitable sample size. If any GW had too less samples to develop RI, three or four GWs were combined for convenient resampling process. The new category through this combination was nominated as gestational period (GP). The resampling process does not simply increase the sample size by re-extracting identical values, but rather by obtaining the arithmetic *x*-values of randomly selected *n* results and iterating the entire previous process *t* times to create a completely different sample. Three hyper-parameters were required for the resampling process: the number of randomly selected samples, arithmetic values as new data, and iteration time. The number of randomly selected samples was set to two and the arithmetic value was defined as the mean value. The mean of two randomly extracted SCr values was calculated from the entire set of SCr measurements within a single GP. This calculation was iterated 120 times, and the distribution of the resulting 120 values needed to satisfy the following Gaussian distribution criteria for the subsequent validation process^13^: (1) kurtosis, 3 (2.5–3.5); (2) skewness, 0 (− 0.5 to 0.5); (3) normality, P-value ≥ 0.05 at Shapiro–Wilk normality test. If the resampling results satisfied a Gaussian distribution, the range composed of the 3rd and 118th values was defined as the 95% CI. Ultimately, if 95% or more of the entire set of SCr measurements contained within a single GP were encompassed by this range, the resampling results were confirmed as the reference results for setting the RI. Computational statistics and graphics were performed using R language and environment for statistical computing version 4.3.2 (R Foundation for Statistical Computing, Vienna, Austria).

### Polynomial regression curve searching

In a study by Harel et al.^14^, it was observed that SCr concentration underwent continuous changes based on GW. As the resampling results were generated using GPs, a polynomial regression curve was employed to establish a continuous RI tailored to different GWs^15^. This approach aimed to capture the ongoing variability in SCr concentrations in relation to GWs. A polynomial regression curve was created using k-fold cross-validation. Because the entire pregnancy duration is commonly divided into trimesters, the value of k was empirically set to 3. The best degree of the polynomial regression curve was explored within the range of 1 to 5, and the degree that yielded the smallest MSE value was selected as the best. The process of exploring the best degree was iterated thousand times, and among the thousand best degrees, the degree that appeared most frequently was selected as the optimal degree. The optimal degree was then used to construct a final polynomial regression curve.

### Renal hyperfiltraion from SCr measurement

Over 70 formulas for estimating GFR were evaluated, and large errors in these formulas did not improve over 60 years; additionally, GFR increases during pregnancy due to absolute hyperfiltration^16,17^. Most modern formulas include age as a variable^16^; however, we considered the GW as important variable. As renal hyperfiltration increases, SCr concentration decreases. In this study, we focused on determining the value of renal hyperfiltration SCr concentration according to GW as a marker of gestational eGFR. The prefix “hyper” indicated, a reference baseline had to be set to calculate renal hyperfiltration. Two distinct concepts were incorporated in the calculation of renal hyperfiltration. The concept of renal hyperfiltration during pregnancy can be easily described as the ratio of SCr measurements during pregnancy to non-pregnancy SCr values. This concept led to the circumstance that renal hyperfiltration remained the same as long as SCr measurements were identical regardless of the timing of the measurement. Thus, comparing the reduction in SCr concentration during pregnancy to the pre-implantation status enabled the prediction of physiological renal hyperfiltration that occurred during pregnancy. If the RIs of pregnant women exhibit linearity, this situation is correct. However, because the GW-specific SCr RI showed curvilinearity, an adjustment was necessary for the calculation of actual pregnancy hyperfiltration. Usually, pregnancy was recognized through changes in the menstrual cycle, so research on physiological changes in the very early stages of pregnancy was scarce. Kapraun et al*.*^18^ used empirical models to predict various physiological parameters of pregnant women across all gestational ages. Obstetricians of our research team generally considered an implantation to occur around GW 4, and various predictive models confirmed the absence of blood flow to the placenta up to GW 4^18^. Based on this, the median GW 0–3 RI was set as the reference baseline SCr concentration (BSC). First, the hyperfiltration gap was calculated as the difference between the BSC and SCr measurements. If the SCr measurements were smaller than the BSC, positive renal hyperfiltration was indicated. As a lower SCr level resulted in more renal hyperfiltration, a gap was defined as follows:

1 $$Hyperfiltration\, gap =BSC- SCr \,measurement$$

Second, a simple hyperfiltration gap ratio was obtained by calculating the ratio of the hyperfiltration gap to BSC.

2 $$Simple \,hyperfiltration \,gap\, ratio =\frac{BSC-SCr\, measurement}{BSC}$$

Third, to obtain the overall hyperfiltration ratio from a simple hyperfiltration gap ratio, 1 was added to represent the change from the original BSC value.

3 $$Overall \,hyperfiltration \,ratio =1+\frac{BSC- SCr \,measurement}{BSC}=1+\frac{BSC}{BSC}-\frac{SCr \,measurement}{BSC}=1+1-\frac{SCr\, measurement}{BSC}=2-\frac{SCr\, measurement}{BSC}$$

### eGFR based renal hyperfiltration

The overall hyperfiltration ratio was not a unit of eGFR used in actual clinical practice, the normal GFR value for non-pregnant women was multiplied by the overall hyperfiltration ratio to convert it into a familiar form.

4 $$Simple\, eGFR \,by\, hyperfiltration =overall \,hyperfiltration \,ratio\times normal\, GFR$$

Harel et al*.*^14^ reported that the median SCr values differed for each GW with curvilinearity. To consider curvilinearity, a correction constant k (*k*_*gw*_) that adjusted a simple eGFR by hyperfiltration based on GW was multiplied. *k*_*gw*_ was defined as the ratio of the median SCr value according to GW to BSC.

5 $$Real\, eGFR \,by \,hyperfiltration =simple\, eGFR \,by\, hyperfiltration\times {k}_{gw}=simple \,eGFR\, by\, hyperfiltration\times \frac{BSC}{median \,SCr\, value\, according \,to\, GW}$$

Finally, the gestational eGFR formula was completed by organizing all equations as follows.

6 $$Gestational \,eGFR\, formula=Organized \,real \,eGFR\, by\, hyperfiltration=simple\, eGFR\, by \,hyperfiltration\times \frac{BSC}{median\, SCr\, value\, according\, to \,GW}=overall \,hyperfiltration ratio\times normal \,GFR\times \frac{BSC}{median \,SCr \,value\, according \,to\, GW}=\left(2-\frac{SCr\, measurement}{BSC}\right)\times normal \,GFR\times \frac{BSC}{median \,SCr \,value \,according \,to\, GW}$$

In consequently, values had to be found were BSC, normal GFR, and median SCr value according to GW. Fortunately, the median SCr value according to GW was obtained from the polynomial regression curve at GW-specific RIs.

### Ethics

This study was conducted retrospectively using existing medical records, and the requirement for obtaining written consent from patients was waived due to the very low risk posed by such research to the patients. This decision was confirmed by the Institutional Review Board (IRB) of WSCH. This study was conducted in accordance with the principles of the Declaration of Helsinki. The protocol of this research was approved by an independently constituted ethics committee within the institution (CR321084).

## Results

### Establishment of RI from the resampling process

**Figure 2:**
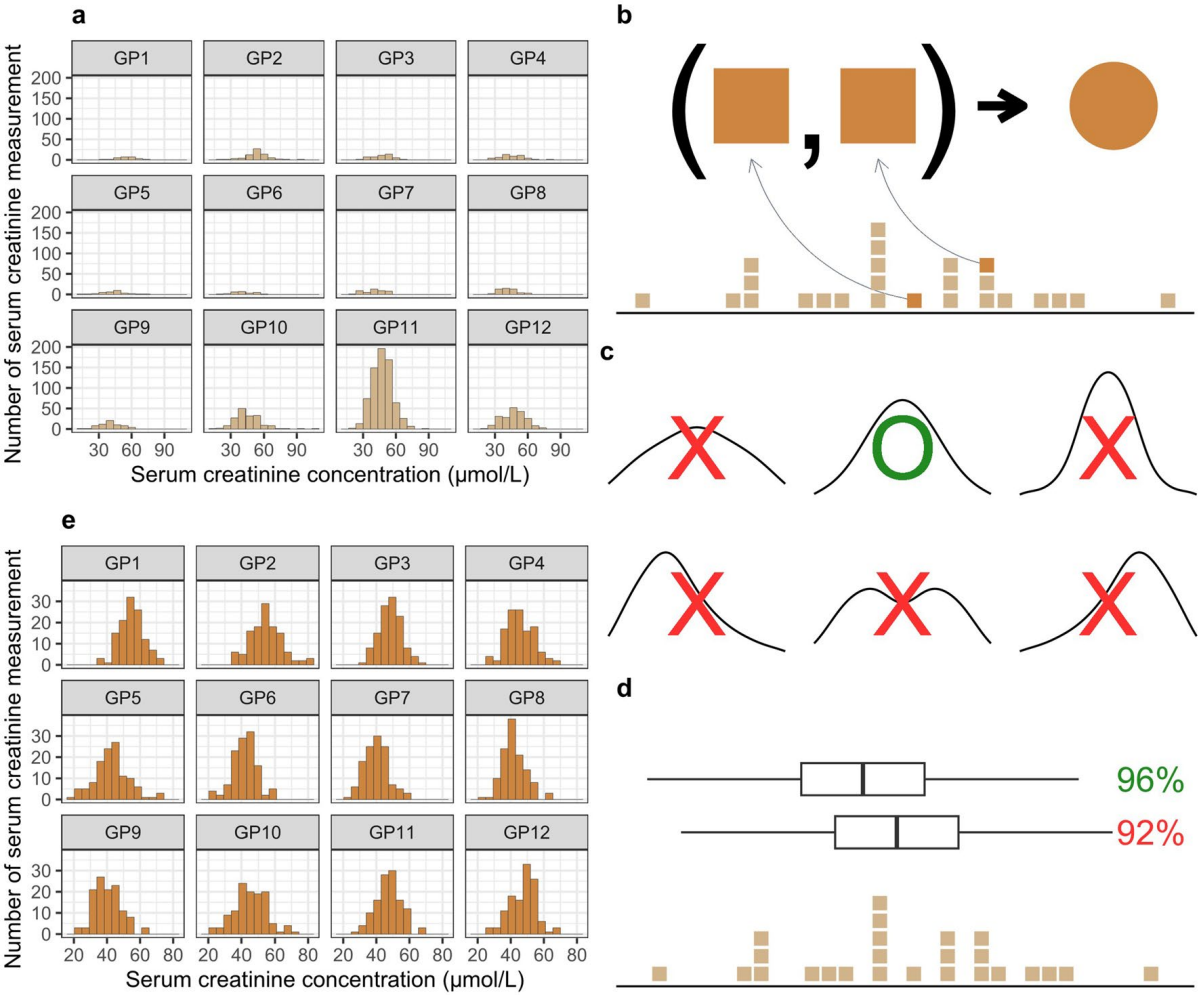
Generating new data using bootstrap resampling. (**a**) Histogram of original SCr concentration according to GP. Although the number of SCr measurements in the first and second trimesters of pregnancy was insufficient, 12 GPs were constructed. However, there was a significant discrepancy in the number of SCr measurements comprising each GP, and their distributions also vary widely. (**b**) Resampling method: resampling was performed separately for each GP. Resampling involved iteratively calculating the average of two randomly selected SCr measurements, and was repeated 120 times. (**c**) Validation of distribution: new data composed of 120 resampled results was validated for adherence to a Gaussian distribution. The kurtosis and skewness were required to be approximately 3 and 0, respectively, while also exhibiting normality. (**d**) Verification of 95% confidence interval (CI) of new data: 95% CI were determined by the 2.5th and 97.5th percentiles for new data that conformed to a Gaussian distribution. The new data, containing over 95% of the original data within its 95% CI range, was ultimately utilized to construct the RI. (**e**) New data for constructing SCr RI: the finalized new data for each GP exhibit a uniform distribution across the resampled results.

We attempted to establish GW-specific SCr RIs; however, among GWs from 0 to 41, only three had more than 120 measurements each (Supplementary Fig. S2A). Nevertheless, despite the recommendation to use a minimum of 20 individuals for RI construction, there were very few GWs with > 20 measurements in the remaining 1st and 2nd trimesters, as most prenatal examinations were in the 3rd trimester (Supplementary Fig. S2A). There were limitations in establishing the RI based solely on the initial measurements (Supplementary Fig. S2B). Because of the prevalence of GWs with fewer than 20 measurements, several GWs were combined to create a GP (Supplementary Fig. S3A). Among the 12 GPs, only one GP satisfied a Gaussian distribution (Supplementary Fig. S3B). However, because the distribution of the 12 GPs was highly diverse (Fig. 2A), they had to be homogenized. The iteration time was set to 120 (Fig. 2B). A new dataset generated through the resampling process must satisfy three key characteristics to be ultimately utilized to establish the RI. First, a new dataset must satisfy normality (Fig. 2C). Normality tests were performed using the Shapiro–Wilk test. Second, kurtosis and skewness were adjusted to approximately 3 and 0, respectively, by rounding (Fig. 2C). The 95% CI was defined as the range between the 2.5th percentile and 97.5th percentile of the new dataset. The 2.5th percentile and 97.5th percentile of the 120 new values were the 3rd and 118th percentiles, respectively^7^. Third, the established RI was verified according to the CLSI recommendation, which requires at least 20 normal samples in the clinical laboratory, with 95% or more falling within the RI^7^ (Fig. 2D). The newly generated GP consisted of more than 20 measurements (Supplementary Fig. S3A), all of which were performed on healthy pregnant women. The measurements included in the GP were utilized through a resampling process to construct and simultaneously verify the RI. Finally, the RIs of the 12 GPs exhibited a Gaussian distribution with a uniform shape (Fig. 2E).

### Optimal polynomial regression curve search

**Figure 3:**
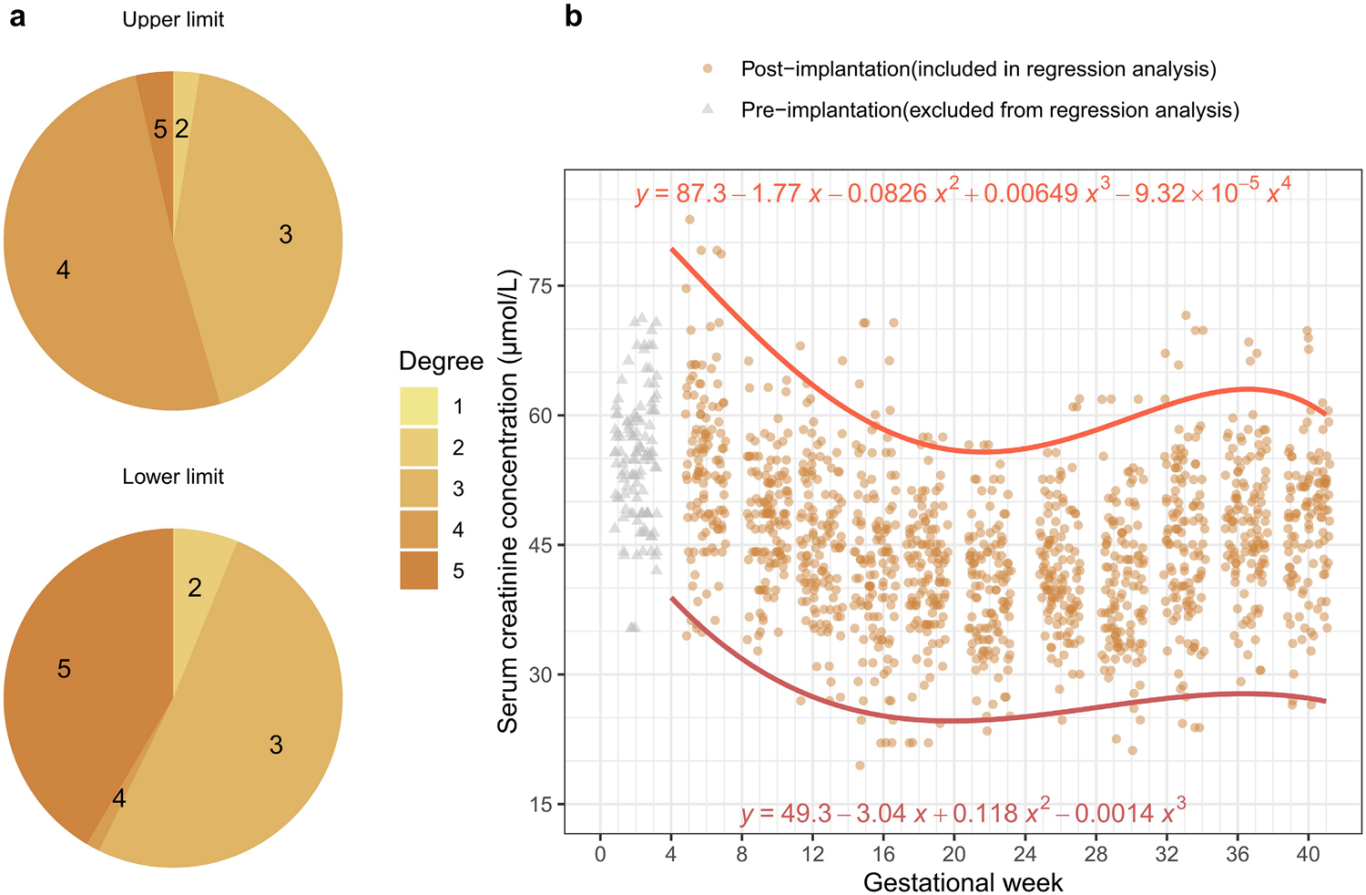
Polynomial regression for GW-specific SCr RI construction. (**a**) Frequency for determining the best degree for polynomial regression when transforming data from GP to GW format. The GP format new data exhibit curvilinearity. Therefore, polynomial regression was conducted on the 97.5th and 2.5th percentiles for determining upper and lower limits, respectively. The method to ascertain the best degree involved calculating the mean squared error (MSE) and employed an empirical approach with threefold cross-validation ranging from 1 to 5. The process for determining the best degree was repeated one thousand times, and the degree that appeared most frequently in the results was chosen as the optimal degree. The optimal degree for the upper and lower limits were 4 and 3, respectively. (**b**) Confirmed optimal polynomial regression curves for upper and lower limits of SCr. The pre-implantation period (0–3 GW, which is 1 GP) was excluded from the regression analysis. The x-value of the polynomial regression curve equations is GW, while the y-value represents SCr values.

Although RI construction was not possible for each individual GW, the twelve constructed RIs based on GP had a pattern similar to the original SCr measurements (Supplementary Fig. S4A,B). To identify the best degree of the polynomial regression curve, the mean square error (MSE) was empirically calculated for five degrees, ranging from 1 to 5: The regression process involved a k-fold cross-validation to determine the optimal degree with the smallest MSE value. This process was iterated 1,000 times, and the degree with the highest frequency was selected as the optimal degree. The 97.5th and 2.5th percentiles of the new datasets for each GP were used to create the upper and lower limits of GW-specific RIs. The optimal degrees of the polynomial regression curve for the upper and lower limits of GP-specific SCr RIs were 4 and 3, respectively (Fig. 3A). Polynomial regression curve equations for the upper and lower limits were used to obtain the exact RI per GW (Fig. 3B, Table 1). One important consideration was that the first GP, representing GW 0–3, was assumed to have no hemodynamic changes, as it corresponded to the pre-implantation period; thus, an RI was not established (gray triangles in Fig. 3B).

**Table 1:** Reference intervals of serum creatinine (SCr) concentration and estimated glomerular filtration rate (eGFR) according to gestational week (GW).

GW	SCr concentration (μmol/L)			eGFR (mL/min)		
	2.5th percentile	97.5th percentile	Median (increment (%)^a^)	2.5th percentile	97.5th percentile	Median (increment (%))
4	38.9	79.3	54.3 (− 1.7%)	72.7	148.6	107.6 (9.0%)
5	36.9	77.1	53.4 (− 3.3%)	72.6	149.7	111.4 (12.9%)
6	35.0	75.0	52.4 (− 5.2%)	73.9	152.2	115.8 (17.3%)
7	33.3	72.9	51.3 (− 7.1%)	76.4	155.8	120.7 (22.3%)
8	31.8	70.8	50.2 (− 9.1%)	79.9	160.2	125.9 (27.6%)
9	30.5	68.8	49.1 (− 11.1%)	84.1	165.3	131.3 (33.1%)
10	29.3	66.9	48.0 (− 13.1%)	88.8	170.8	136.8 (38.6%)
11	28.3	65.1	46.9 (− 15.1%)	93.9	176.6	142.3 (44.2%)
12	27.4	63.4	45.8 (− 17.1%)	99.1	182.5	147.6 (49.6%)
13	26.6	61.9	44.8 (− 18.9%)	104.2	188.3	152.6 (54.6%)
14	26.0	60.6	43.9 (− 20.5%)	109.3	193.9	157.4 (59.5%)
15	25.5	59.4	43.0 (− 22.2%)	114.1	199.2	161.7 (63.9%)
16	25.1	58.3	42.2 (− 23.6%)	118.5	204.0	165.6 (67.8%)
17	24.8	57.4	41.6 (− 24.7%)	122.4	208.3	169.0 (71.3%)
18	24.6	56.7	41.0 (− 25.8%)	125.7	212.0	171.8 (74.1%)
19	24.5	56.2	40.5 (− 26.7%)	128.5	215.0	174.0 (76.3%)
20	24.5	55.9	40.2 (− 27.2%)	130.5	217.2	175.6 (77.9%)
21	24.5	55.7	40.0 (− 27.6%)	131.9	218.7	176.6 (79.0%)
22	24.6	55.7	39.8 (− 28.0%)	132.6	219.5	177.0 (79.4%)
23	24.8	55.8	39.8 (− 28.0%)	132.5	219.4	176.8 (79.2%)
24	25.0	56.0	39.9 (− 27.8%)	131.8	218.5	176.0 (78.4%)
25	25.2	56.4	40.1 (− 27.4%)	130.3	216.9	174.6 (76.9%)
26	25.4	56.9	40.5 (− 26.7%)	128.3	214.6	172.7 (75.0%)
27	25.7	57.5	40.9 (− 26.0%)	125.7	211.6	170.4 (72.7%)
28	26.0	58.2	41.4 (− 25.1%)	122.7	208.0	167.6 (69.8%)
29	26.2	58.9	41.9 (− 24.2%)	119.3	203.9	164.4 (66.6%)
30	26.5	59.6	42.6 (− 22.9%)	115.6	199.4	161.0 (63.2%)
31	26.8	60.3	43.2 (− 21.8%)	111.8	194.6	157.5 (59.6%)
32	27.0	61.0	44.0 (− 20.4%)	108.0	189.7	153.8 (55.9%)
33	27.2	61.6	44.7 (− 19.1%)	104.4	184.7	150.2 (52.2%)
34	27.3	62.2	45.4 (− 17.8%)	101.1	179.9	146.8 (48.8%)
35	27.4	62.6	46.1 (− 16.6%)	98.4	175.5	143.6 (45.5%)
36	27.5	62.8	46.8 (− 15.3%)	96.4	171.5	140.9 (42.8%)
37	27.4	62.8	47.4 (− 14.2%)	95.3	168.3	138.7 (40.6%)
38	27.4	62.6	47.9 (− 13.3%)	95.5	166.0	137.3 (39.1%)
39	27.2	62.0	48.4 (− 12.4%)	97.1	164.9	136.8 (38.6%)
40	26.9	61.1	48.6 (− 12.0%)	100.5	165.2	137.4 (39.2%)
41	26.5	59.8	48.7 (− 11.9%)	105.9	167.3	139.3 (41.2%)

^a^Increment (%) refers to the ratio of change in SCr and eGFR during the pre-implantation period (0–3 GW).

### Verification of GW-specific SCr RIs

Although it is known that the SCr concentration typically decreases during pregnancy, the precise RI for this phenomenon has not been established. Because this study did not construct RIs using traditional or classical methods, verification is essential. For verification, we utilized the results of Harel et al.^14^, which directly measured SCr concentrations in approximately 244,000 Canadian pregnant women. Harel et al.^14^ periodically measured SCr concentrations during pregnancy and reported mean values from the 95th, 75th, and 50th percentiles. Following the method used to determine the upper and lower limits of GW-specific SCr RIs, optimal polynomial regression curves were derived at the 95th, 75th, and 50th percentile values of the new dataset (Supplementary Fig. S5A). The average differences among the 95th, 75th, and 50th percentiles were − 5.0%, − 2.7%, and − 2.5%, respectively. The GW with the smallest difference is GW 10, followed by GW 8. The second trimester demonstrated the largest difference; however, when analyzed by GW, GW 40 exhibited the largest difference (Table 2, Supplementary Fig. S5B).

**Table 2:** Comparison of serum creatinine concentration (μmol/L) according to gestational week (GW) between the actual measurement studies and our study.

GW	Trimester	95th percentile			75th percentile			50th percentile			Mean difference by GW	Difference by trimester
		Harel et al.	This study	Difference	Harel et al.	This study	Difference	Harel et al.	This study	Difference		
4	1	75	71.8	− 3.2	64	61.0	− 3.0	58	54.3	− 3.7	− 3.3	− 1.5
6	1	70	67.3	− 2.7	60	58.5	− 1.5	54	52.4	− 1.6	− 1.9	
8	1	65	63.6	− 1.4	56	55.8	− 0.2	50	50.2	0.2	− 0.5	
10	1	61	60.6	− 0.4	53	53.2	0.2	48	48.0	0.0	− 0.1	
12	1	61	58.2	− 2.8	52	50.7	− 1.3	47	45.8	− 1.2	− 1.8	
14	2	59	56.3	− 2.7	51	48.5	− 2.5	46	43.9	− 2.1	− 2.4	− 4.1
16	2	59	55.0	− 4.0	50	46.8	− 3.2	45	42.2	− 2.8	− 3.3	
18	2	58	54.1	− 3.9	49	45.6	− 3.4	45	41.0	− 4.0	− 3.8	
20	2	59	53.6	− 5.4	50	44.9	− 5.1	45	40.2	− 4.8	− 5.1	
22	2	58	53.5	− 4.5	50	44.8	− 5.2	44	39.8	− 4.2	− 4.6	
24	2	59	53.6	− 5.4	49	45.2	− 3.8	45	39.9	− 5.1	− 4.8	
26	2	59	54.0	− 5.0	50	46.1	− 3.9	45	40.5	− 4.5	− 4.5	
28	3	59	54.7	− 4.3	50	47.3	− 2.7	44	41.4	− 2.6	− 3.2	− 4.0
30	3	59	55.4	− 3.6	50	48.8	− 1.2	44	42.6	− 1.4	− 2.1	
32	3	60	56.2	− 3.8	50	50.3	0.3	45	44.0	− 1.0	− 1.5	
34	3	63	57.1	− 5.9	52	51.7	− 0.3	46	45.4	− 0.6	− 2.3	
36	3	66	58.0	− 8.0	54	52.7	− 1.3	48	46.8	− 1.2	− 3.5	
38	3	70	58.7	− 11.3	57	53.1	− 3.9	50	47.9	− 2.1	− 5.8	
40	3	76	59.4	− 16.6	61	52.6	− 8.4	53	48.6	− 4.4	− 9.8	

### Gestational eGFR formula from physiological SCr hyperfiltration

**Figure 4:**
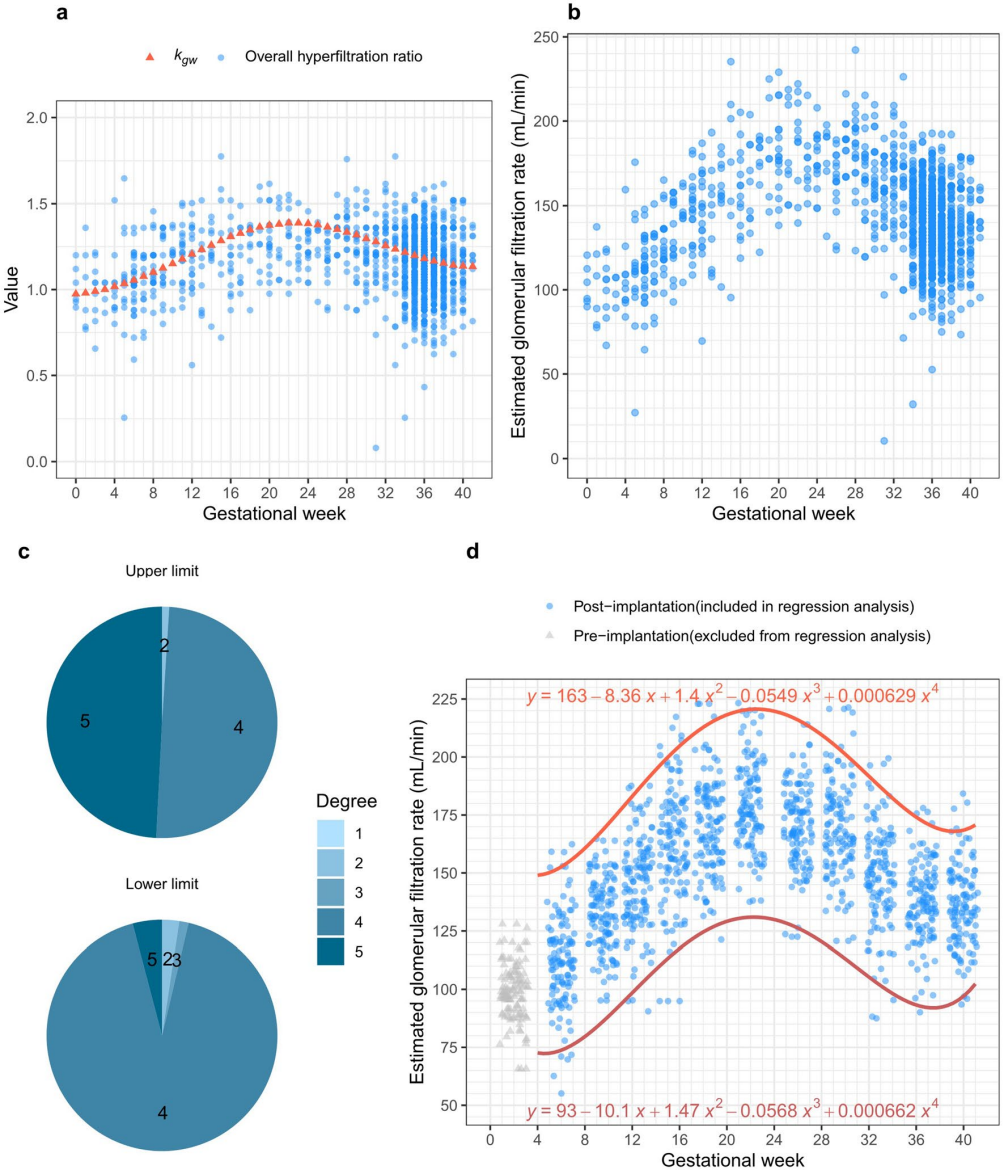
Derived eGFR from serum creatinine SCr hyperfiltration and RI construction. (**a**) Two values for eGFR calculation. Pregnancy-related hyperfiltration can be inferred from decreased SCr values compared to non-pregnant states (Eq. (3), overall hyperfiltration ratio). However, since SCr based on GW exhibited curvilinearity, a correction factor (*k*_*gw*_) was applied for each GW in the overall hyperfiltration ratio. (**b**) Derived eGFR from original SCr measurement. The overall hyperfiltration ratio and the *k*_*gw*_ were substantiated for deriving eGFR from SCr measurements, and the final calculation process is defined as gestational eGFR formula (Eq. 6). (**c**) Best degrees for eGFR RI. Similar to the process of constructing SCr RI, the best degrees of the upper and lower limit for eGFR RI were explored a thousand times, respectively, and were both 4. (**d**) Confirmed optimal polynomial regression curves for the upper and lower limits of eGFR. The pre-implantation period (0–3 GW, which is 1 GP) was also excluded from the regression analysis.

As maternal body fluid significantly increases during pregnancy and impacts body weight more than BSA, it is difficult to apply the present eGFR formula to pregnant women, as it inherently incorporates BSA. Ma et al.^19^ directly measured the GFR of healthy Chinese adults using technetium 99m-diethylenetriamine penta-acetic acid, and found a mean value of 110.1 mL/min/1.73 m^2^ for women below 50 years old. Considering the greater increase in body fluids than in BSA during pregnancy, using a GFR calculation that does not consider BSA is preferable^4^. The mean BSA of women younger than 50 years in Korea, measured using the alginate method^20^, was 1.62 m^2^, which was then multiplied by 103.1 mL/min. Finally, this value was multiplied by the overall hyperfiltration ratio to generate eGFR, accounting for hyperfiltration. The median polynomial regression curve was 56.7 − 0.223 $\times$ GW − 0.113 $\times$ GW^2^ $+$ 0.00545 $\times$ GW^3^ − 0.0000653 $\times$ GW^4^, and the BSC was 55.25 μmol/L. As the median polynomial regression curve and BSC were developed for *k*_*gw*_ (Fig. 4A), final formula was as follows.

7 $$Gestational \,eGFR formula =\left(2-\frac{SCr\, measurement (\upmu {\text{mol}}\text{/}{\text{L}})}{55.25 \, \upmu {\text{mol}}\text{/}{\text{L}}}\right)\times {103.1 \, {\text{mL}}\text{/}{\text{min}}}\times \frac{55.25 \, \upmu {\text{mol}}\text{/}{\text{L}}}{56.7-0.223\times GW-0.113 \times {GW}^{2}+0.00545 \times {GW}^{3}-0.0000653\times {GW}^{4}}$$

The original SCr measurements were transferred to eGFRs using the formula (Fig. 4B). The eGFR values calculated using the gestational eGFR formula were used to construct GW-specific eGFR RIs in the same manner as those used to establish GW-specific SCr RIs (Fig. 4C,D). The GW-specific eGFR RI values are shown in Table 1.

## Discussion

Both GFR and renal plasma flow increase during pregnancy. Serial observations suggest that the GFR progressively peaks in mid-pregnancy and remains constant thereafter^21,22^. On average, the GFR during the second half of pregnancy exceeds non-gravid levels by 40–50%^21^. Accurate identification and management of these physiological changes through periodic prenatal care is crucial, as failure to do so poses risks to both the woman and developing fetus. Park et al.^23^ reported that the degree of renal hyperfiltration in midterm pregnancy was associated with adverse pregnancy outcomes, whereas Harel et al.^24^ reported that decreased glomerular hyperfiltration in early pregnancy increased the probability of pre-term birth. This study aimed to develop a formula predicting normal gestational eGFR rather than investigating the association between eGFR and pregnancy adverse outcomes. Despite the limited number of results derived from normal pregnant women, the observed increase in eGFR up to GW 23, particularly during the mid-trimester, suggests no major flaws in the design of this study.

The bootstrap resampling method employed in this study was not necessarily the optimal approach for augmenting sample size. If the distribution does not have a small number of measurements or exhibits extreme skewness, the bootstrap method can be applied as a non-parametric approach to calculate a 95% CI^25^. When the skewness was greater than or equal to 4, the effectiveness of the bootstrap method decreased^25^. However, because all 12 GPs had skewness values below 1.5 (Supplementary Fig. S3B), the bootstrap method was employed to amplify the reference data to satisfy the second recommendation of the CLSI. By employing the non-parametric bootstrap, uncertainty was estimated through an empirical approximation of the sampling distribution. This entailed resampling from the initial data with replacement, while maintaining the original structure of the data^25^.

Women with renal disorders experience several problems during pregnancy due to the increased physiological changes associated with renal dysfunction, risk of disease progression, potential teratogenicity of medications, and increased risk of developing complications such as preeclampsia and preterm delivery^23,24,26^. Although the prevalence of CKD in women of childbearing age is relatively low (0.1–4%), CKD significantly increases the risk of adverse maternal and perinatal outcomes^26^. Pregnancy-related acute kidney injury (AKI) is a common cause of AKI in young women and is associated with future risks of CKD, hypertension, and cardiovascular diseases^27^.

Therefore, accurate evaluation of renal function during pregnancy is crucial. However, Smith et al.^5^ suggested that the sole application of SCr concentration or SCr-based equations tend to substantially underestimate renal function during pregnancy. Park et al.^23^ reported that the absence of prominent midterm renal hyperfiltration, marked by an extremely high eGFR, may be a significant risk factor for poor pregnancy outcomes in women without functional renal impairment. Harel et al.^24^ reported that blunted glomerular hyperfiltration during early pregnancy may be associated with an increased risk of pre-term birth and perinatal mortality. Kang et al.^11^ demonstrated that the SCr concentration could predict the risk of adverse pregnancy outcomes and the number of co-occurring adverse pregnancy outcomes. When using the GW-specific SCr distribution, the predictive power of adverse pregnancy outcomes was more robust based on beta coefficients and their *p-*values compared to the raw SCr distribution^11^.

The commonly used mMDRD formula is inaccurate for predicting GFR in pregnant women because the MDRD Formula, which estimates GFR using a combination of serum markers and clinical parameters, is the standard clinical method for assessing renal function in patients with CKD^5,6,28,29^. While GFR increases by approximately 40% during pregnancy compared to that in non-pregnant women^1^, the overall increment in eGFR during pregnancy in this study was approximately 53.8%. The eGFR calculated using our formula had a distribution similar to that calculated using GP with midterm hyperfiltration, indicating its utility in predicting eGFR during pregnancy.

Determining the RI in pregnant women remains challenging because of the continuous changes in the physiological, hormonal, and biochemical characteristics of the mother and fetus as pregnancy progresses. Several biochemical markers can be used to assess abnormalities when a pregnancy deviates from the normal course. SCr concentration is mainly used to assess certain maternal conditions, such as renal diseases and preeclampsia. Compared with the results of Harel et al.’s study^12^, the 95th, 75th, and 50th percentile values of SCr RI in this study were generally low, with the 95th percentile exhibiting the greatest difference (Table 2). Owen et al.^30^ reported that the 3rd percentile value of the amniotic fluid index varied among Asians, Pacific Islanders, and non-Hispanic white populations; the non-Hispanic white group exhibited a lower value than the other populations. Since the 3rd percentile of the Asian amniotic fluid index was greater than that of non-Hispanic whites, the relative SCr level would be lower for Asians than for non-Hispanic whites. The results of the present study are consistent with this trend.

Real-time clinical application of the normal RI for SCr concentration and eGFR based on GW can serve as a screening test for two reasons. First, if SCr concentrations and eGFR are lower than those of RIs or show blunted glomerular hyperfiltration, the possibility of pregnancy-induced kidney disease or preeclampsia is implied. Conversely, if the SCr concentration and eGFR are higher than the RIs, severe anemia and/or hypoproteinemia may be suspected. Real-time clinical application of RIs of SCr concentration and eGFR based on GW may help in the early detection of adverse pregnancy outcomes.

Our study was limited because the data were obtained from a single center with limited geographical coverage and, therefore, it could not represent all pregnant women. However, it is undeniable that the significance lies in developing and utilizing the first real-time clinical tool for monitoring renal function of pregnant women from patients in a single institution for decade. While the BSA value organized for developing our formula was directly measured of Korean adult women^20^, the measured GFR values for healthy adult females in Korea were not available, this study relied on a Chinese study^19^ as an East Asian reference. The normal GFR values adapted into our formula may not represent Korean women specifically, but efforts were made to apply values from a racially similar population to minimize errors. Furthermore, this study solely relied on the results of serum samples and did not confirm the absence of renal function abnormalities through actual urine tests of healthy pregnant women. To address this limitation, the authors meticulously analyzed the patients’ underlying diseases and pregnancy adverse outcomes, carefully selecting diseases that could impact renal function. Efforts were made to include only the results of actual healthy pregnant women in the formula construction process. In this study, eGFR was calculated as the percentage of SCr hyperfiltration. The 24-h urine creatinine clearance is the most commonly used GFR test method in clinical practice; however, it is affected by muscle mass, weight, height, and food intake. Collecting a 24-h urine sample throughout pregnancy is impractical because of weight changes throughout gestation^31^. Therefore, measuring a precise GFR in pregnant women, which required examining substances filtered by the glomeruli, was very challenging. Finally, while estimating GFR through cystatin C instead of SCr may be considered more accurately, SCr was used due to its more frequent use in routine prenatal examinations in South Korea. So, it was allowing for a greater number of measurements to be extracted.

We successfully established RIs for SCr concentration and eGFR based on GW among Korean women with normal pregnancies by applying the bootstrap resampling method. While our results were derived through mathematical deduction, these tools assisted in determining eGFR quite easily. Therefore, to accurately evaluate normal RIs for SCr concentration and eGFR in pregnant women, big data analytical studies that represent pregnant women and verification studies using our data are needed.

### Supplementary Information

Supplementary Figures.

## Data Availability

Detailed data supporting the findings and equations of this study are not available because of privacy concerns and hospital regulation restrictions to protect patients. Anonymized data are available with permission from the corresponding author upon reasonable request.

## Code availability

The code utilized for the novel analysis in this manuscript can be accessed publicly on the GitHub page: https://github.com/WCH-AI-LAB/GSR. We used R software version 4.3.2. Additionally, please note that the analysis code requires the “moments” package in R to be installed for proper execution.
