# Supplementary material for: Development of a formula for estimated glomerular filtration rate in pregnant women from physiological hyperfiltration of serum creatinine
Source: Sci Rep. 2024 Mar 27;14:7229. doi: 10.1038/s41598-024-57737-0 (PMC10973345; doi:10.1038/s41598-024-57737-0)

**Supplementary Figures**

**
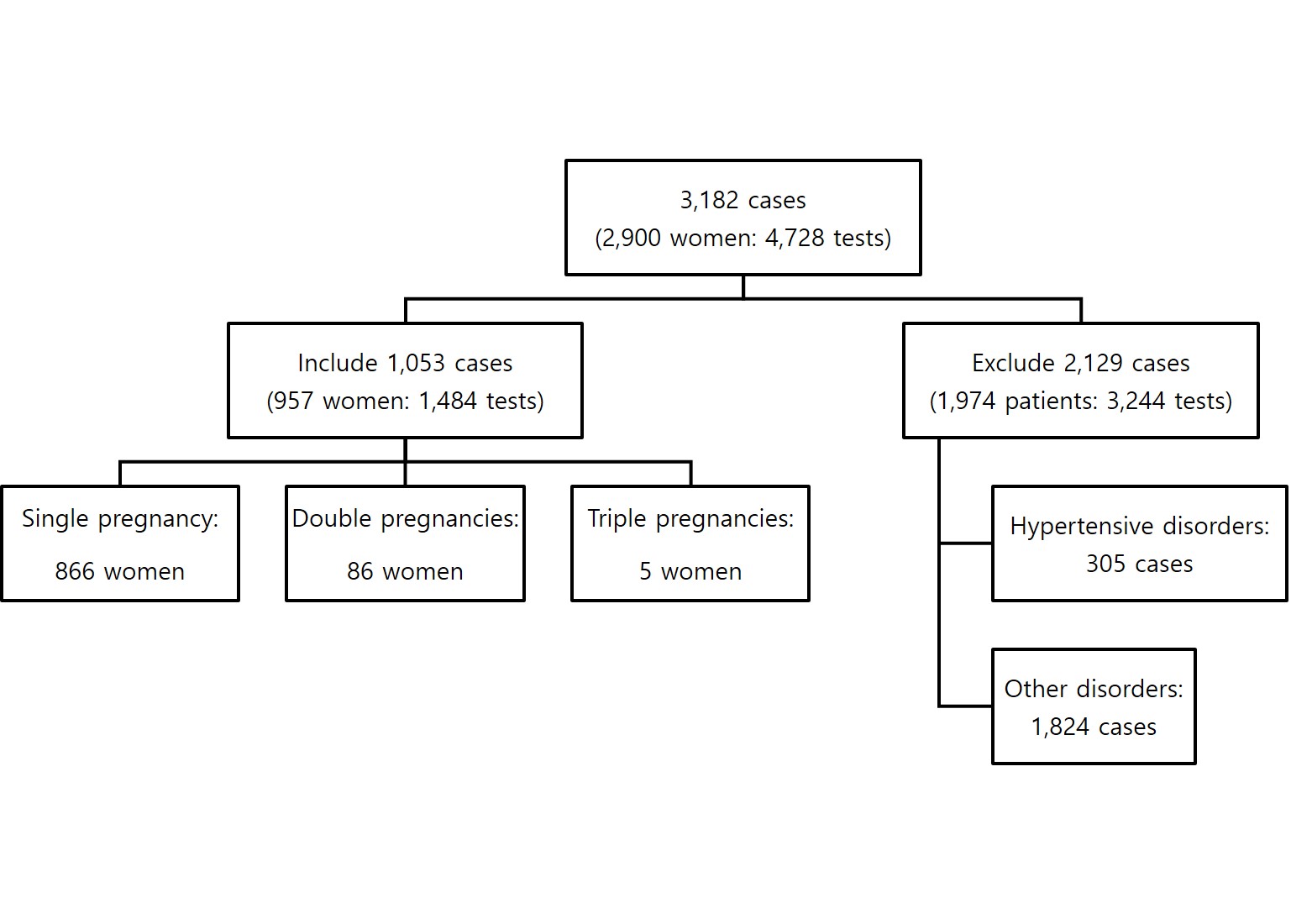
Supplementary Fig. S1. Normal pregnant women in the original data.**

**Supplementary Fig. S2. Original data of serum creatinine (SCr) measurement. a**. Number of SCr measurement according to gestational weeks (GWs). The color of the column varies based on the number of included SCr measurements. Pale blue indicates less than 20. Sky blue represents greater than or equal to 20 and less than 120. Dodger blue means greater than or equal to 120. **b**. Histograms according to GW. The color has the same meaning as depicted in Supplementary Figure 2A. **c**. Verification of GW with more than 20 measurements. GPs that show normality confirmed by the Shapiro−Wilk test are plotted with green circles, while those that do not are plotted with red triangles. The green rectangles satisfying a Gaussian distribution with kurtosis and skewness of 3 and 0, respectively, are depicted. Six-GW (29, 32, 35, 37, 38, and 40) satisfy a Gaussian distribution.


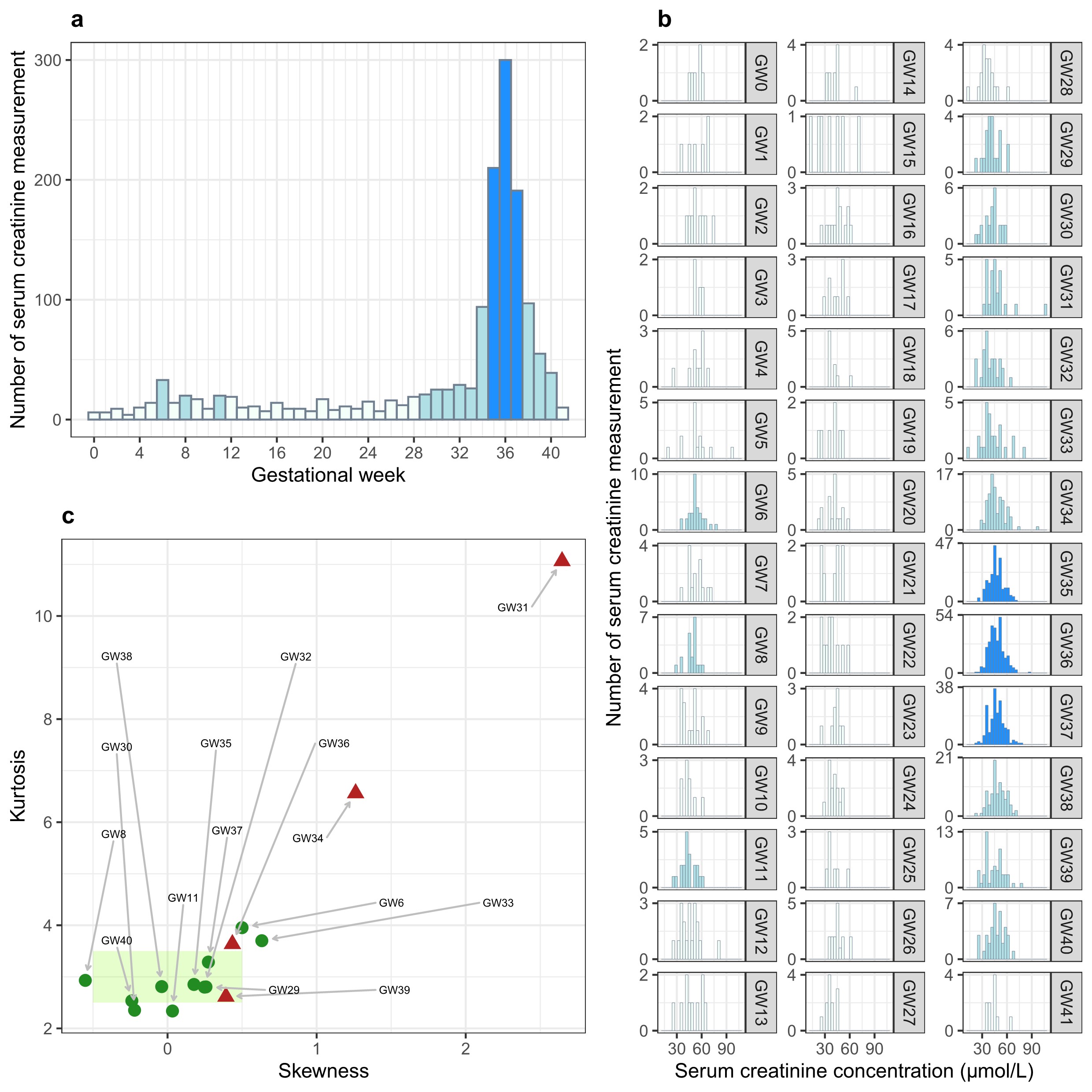


**Supplementary Fig. S3. Gestational period (GP) generation. a**. Information of gestational periods (GPs). GPs formed by combining three or four gestational weeks (GWs) are designed to have serum creatinine measurements exceeding 20. Colors indicate trimesters. **b**. Verification of GPs with a Gaussian distribution. The same plot as Supplementary Figure 2C was utilized. The GP generation developed to eliminate GWs with fewer than 20 measurements still exhibits a variety of distributions.


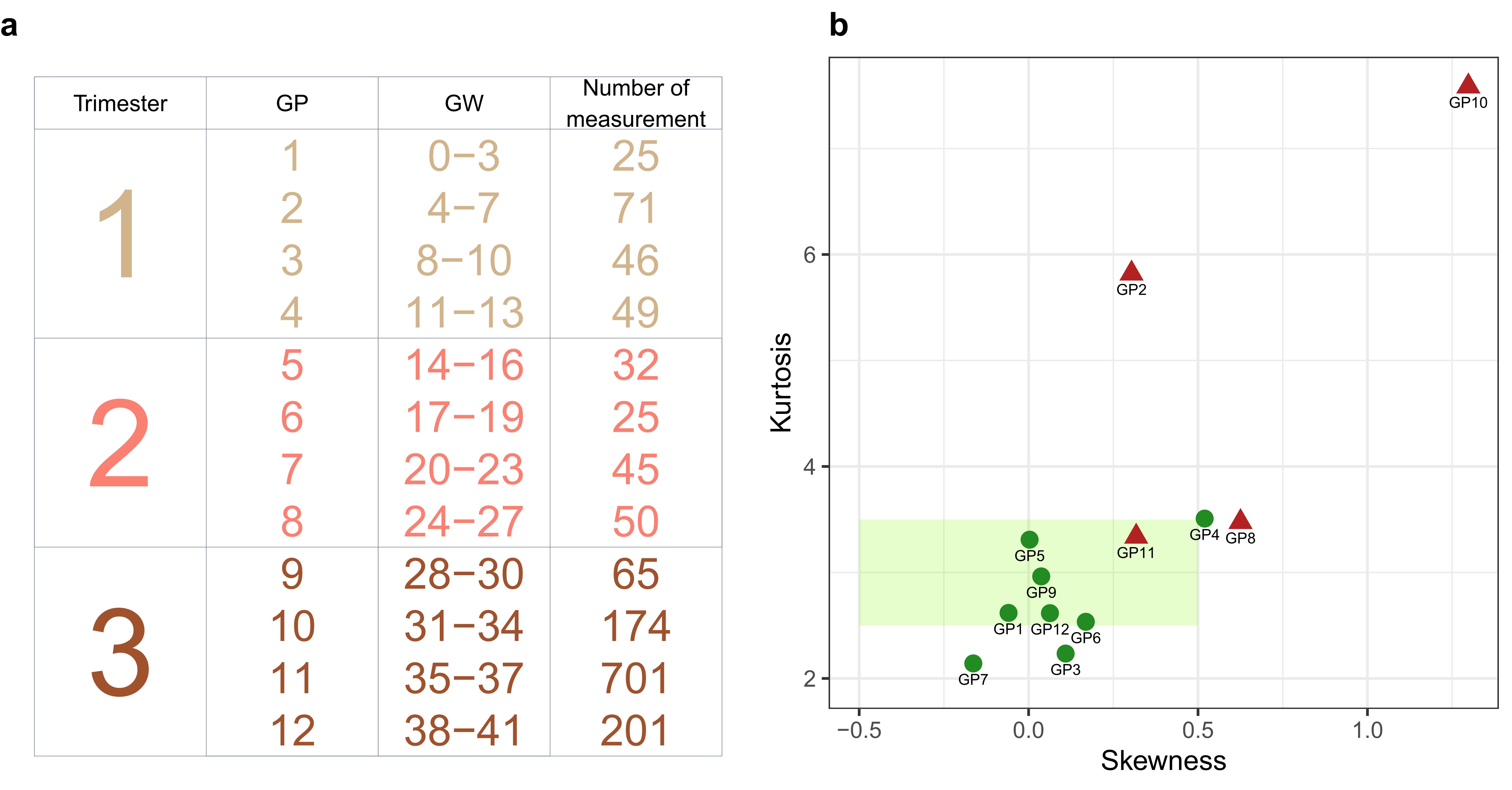


**Supplementary Fig. S4. Effects of the bootstrap resampling method on distribution. a**. Original SCr concentration according to gestational week (GW). SCr concentrations decrease in second trimester, and then increase again near a labor. **b**. New data from bootstrap resampling method. Applying bootstrap resampling method eliminates outliers and maintains the overall distribution shape. Each GP serves as material for constructing reference interval.


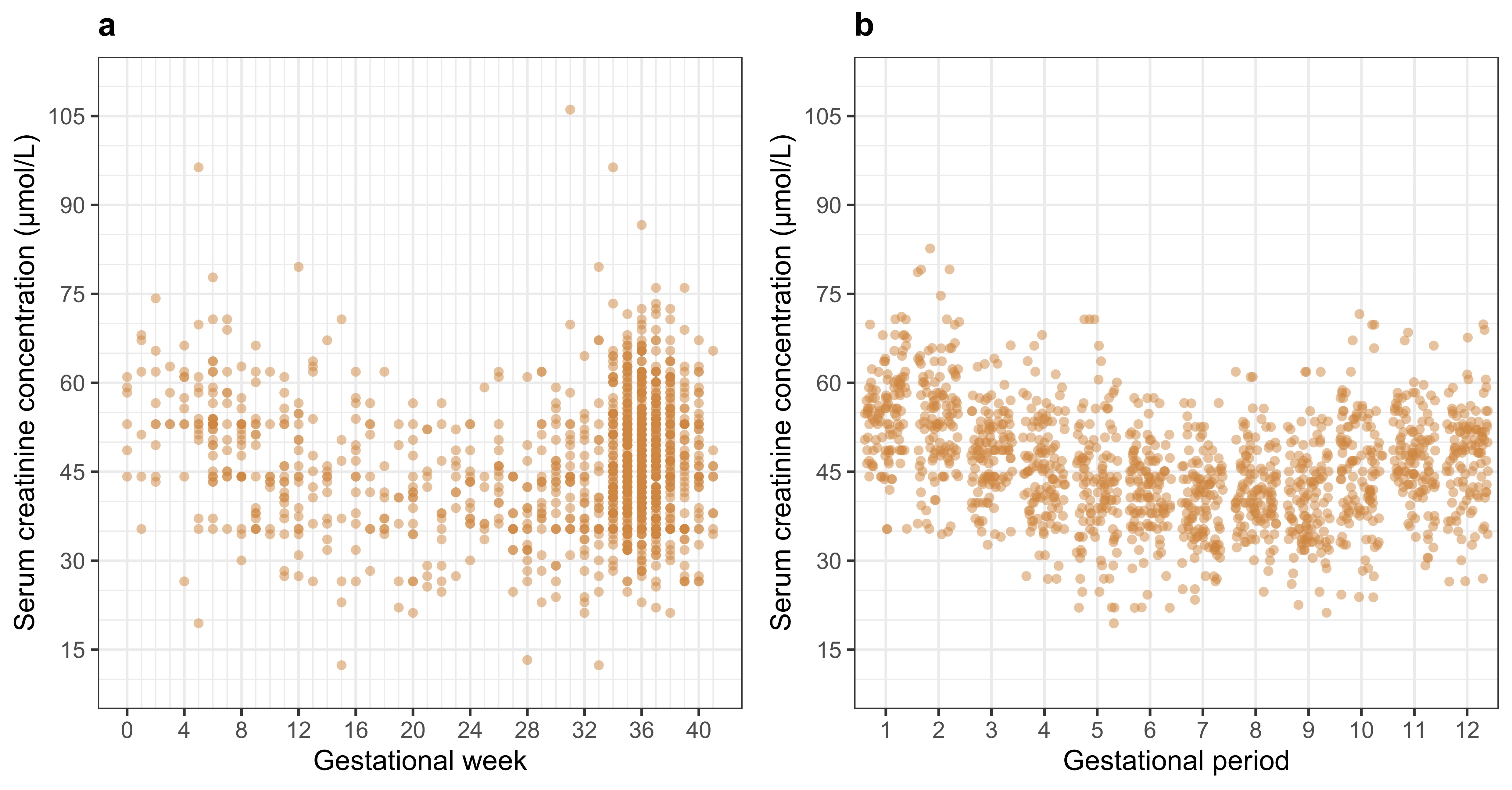


**Supplementary Fig. S5. Validation of the accuracy of the bootstrap resampling method. a**. Best degrees of 95^th^, 75^th^, and 50^th^ percentiles. The process of calculating reference interval (RI) is applied, and three percentiles are subjected to polynomial regression analysis. **b**. Difference between directly measured research results and the polynomial regression curves. While the actual measured values maintain a plateau at minimum point, the results of this study, calculated using a polynomial regression curve, exhibit a concave shape.


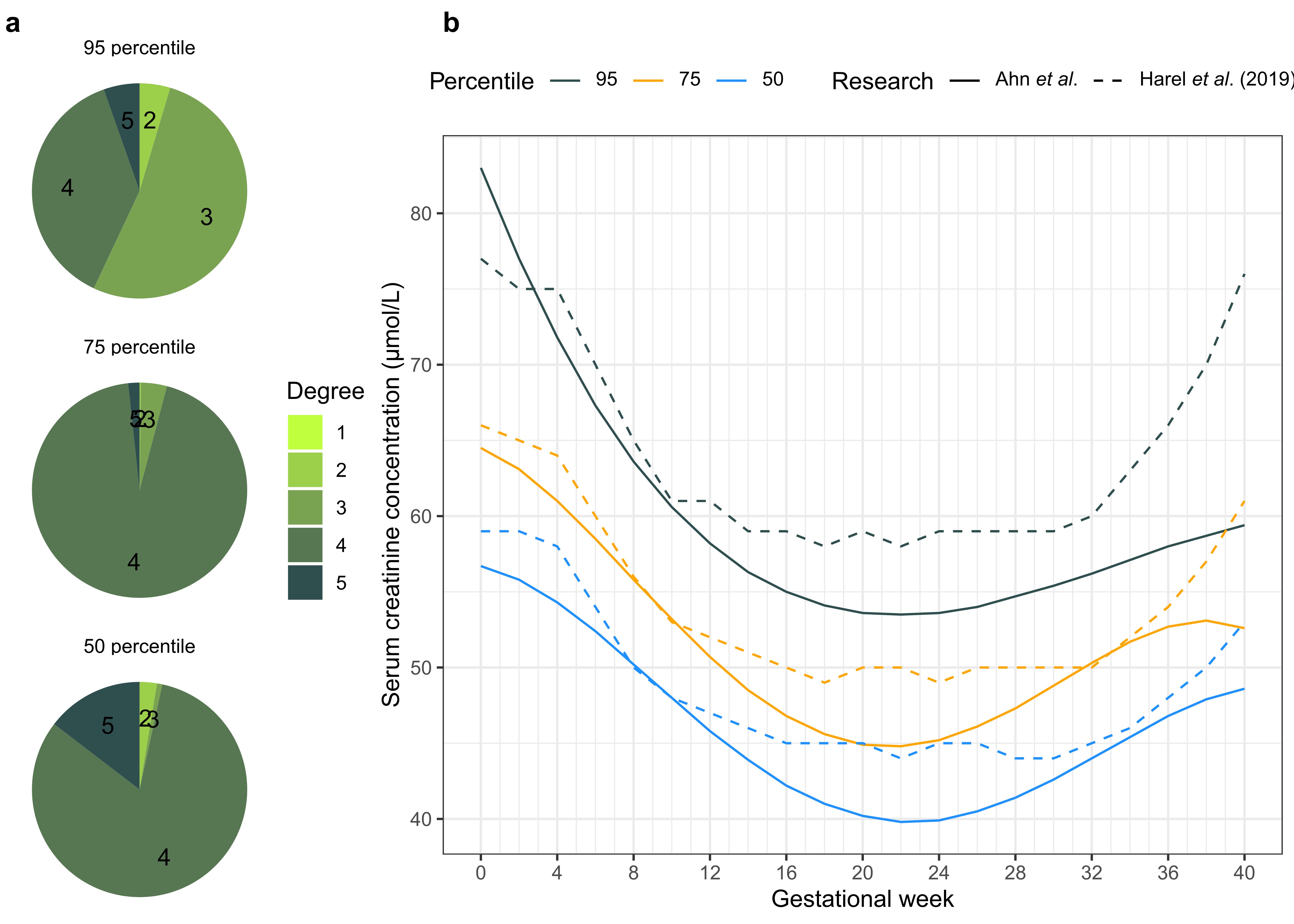

Supplement: Supplementary file 1 — Supplementary Figures. [file 41598_2024_57737_MOESM1_ESM.docx]
